# How can natural products serve as a viable source of lead compounds for the development of new/novel anti-malarials?

**DOI:** 10.1186/1475-2875-10-S1-S2

**Published:** 2011-03-15

**Authors:** Eric Guantai, Kelly Chibale

**Affiliations:** 1 Department of Chemistry, University of Cape Town, Rondebosch, 7701, South Africa; 2 Institute of Infectious Disease and Molecular Medicine, University of Cape Town, Rondebosch, 7701, South Africa

## Abstract

Malaria continues to be an enormous global health challenge, with millions of new infections and deaths reported annually. This is partly due to the development of resistance by the malaria parasite to the majority of established anti-malarial drugs, a situation that continues to hamper attempts at controlling the disease. This has spurred intensive drug discovery endeavours geared towards identifying novel, highly active anti-malarial drugs, and the identification of quality leads from natural sources would greatly augment these efforts. The current reality is that other than compounds that have their foundation in historic natural products, there are no other compounds in drug discovery as part of lead optimization projects and preclinical development or further that have originated from a natural product start-point in recent years. This paper briefly presents both classical as well as some more modern, but underutilized, approaches that have been applied outside the field of malaria, and which could be considered in enhancing the potential of natural products to provide or inspire the development of anti-malarial lead compounds.

## Background

Malaria remains the most devastating tropical disease, with staggering infection and mortality statistics. The WHO World Malaria Report 2008 estimated that there were approximately 247 million malaria cases among 3.3 billion people at risk in 109 countries where malaria is currently considered prevalent; 87% of these cases were reported in the African region. The disease caused nearly one million deaths, 91% of which were in Africa, and 85% of these were of children under 5 years [1]. The main challenge to the effective management of diagnosed malaria cases has been, and continues to be, the resistance of the causative microorganisms to known anti-malarials, which results in the non-resolution of symptoms, recrudescence, and ultimately in treatment failure [2-4].

Various strategies have been embraced in the fight against malaria in general, and anti-malarial drug resistance in particular, and include the improvement of prescribing habits and therapeutic protocols, prevention of infection and the use of combination therapies, with particular emphasis being placed on artemisinin-based combination therapy (ACT) [2,3,5-7]. In tandem with these efforts has been the intensive drug discovery effort aimed at developing new anti-malarial drugs or modifying existing ones, and which targets the identification of novel compounds that exhibit excellent experimental and clinical anti-malarial efficacy without showing any evidence of resistance. Unfortunately the current reality is that other than compounds that have their foundation in historic natural products (such as quinine, artemisinin, hydroxynaphthoquinones, doxycyclin, clindamycin, and azithromycin), there are no other compounds in preclinical development or further that have originated from a natural product start-point in recent years. There are not even any compounds in current anti-malarial lead optimization projects that have come from natural products in recent years. Many natural products have shown potent anti-plasmodial effects but, for a variety of reasons, including chemical tractability issues, these have not been pushed forward into hit-to-lead drug discovery projects.

## Generic approaches to anti-malarial natural product drug discovery

Nature is, as ever, an extremely rich source of potential anti-malarial agents [8], with the anti-malarial drugs quinine and artemisinin being outstanding examples of therapeutic natural products [9,10].

**Figure 1:**
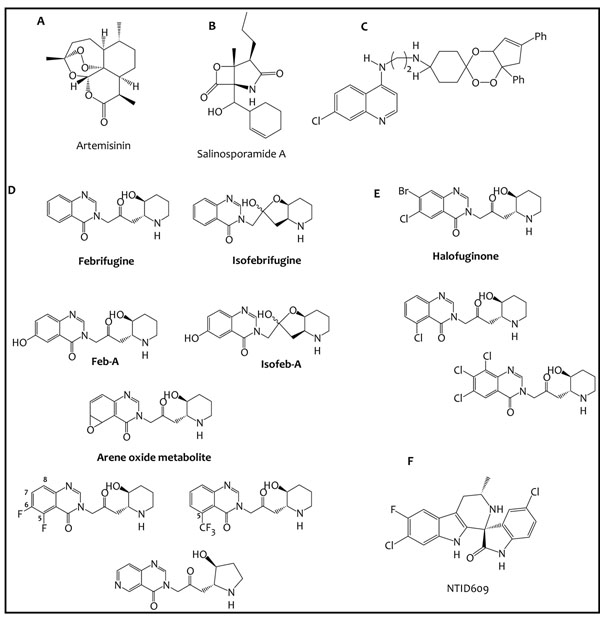
**A:** structure of artemisinin; **B:** salinosporamide A, an antimalarial compound isolated from marine actinomycetes; **C:** an example of a highly active trioxaquine; **D:** febrifugine, isofebrifugine, metabolite feb-A and its synthetic analog isofeb-A, the arene oxide metabolite and examples of potent but less hepatotoxic halogenated febrifugine analogs; **E:** Halofuginone, as well as some highly active febrifugine analogs identified by virtual screening; **F:** NTID609, an extremely active and highly promising anti-malarial spiroindolone.

Following the serendipitous or rational discovery of a biologically active natural material, the conventional approach to natural product development has been the bioassay-guided fractionation of extracts derived from such material, and the subsequent isolation and characterization of pure, active compounds. With artemisinin as an illustrative example (Figure 1) [9-12], the identification of promising compounds in this way usually triggers medicinal chemistry efforts geared towards the total synthesis of the identified compounds and/or the generation of analogs, and which are aimed at providing a supplementary source of the product for further study, revealing structure-activity relationships (SAR), identifying more potent analogs and/or overcoming challenging physicochemical and biological properties. Using comparable approaches, the continuing search for new anti-malarial drugs from natural sources has led to the identification of an impressive range of structurally diverse compounds from a variety of chemical classes [9,13-15].

## Challenges facing conventional natural product drug discovery

The challenges facing conventional anti-malarial natural product drug discovery (and indeed natural product drug discovery in general) are many, and range from the basic problems of reliable access and supply, seasonal or environmental variations in the composition of living organisms and loss of source through extinction or legislation, to the more practical concerns associated with the complexity of the mixtures after fractionation, the isolation of very small quantities of bioactive substance and challenging physicochemical properties such as solubility and stability [16].

Part of the solution may be either to look for ways to supplement the natural sources to meet the existing demand of known bioactive molecules, or to consciously widen the scope of the biodiversity that is sampled for natural product drug discovery by, for example, the exploration of extreme habitats not routinely considered [17,18]. Recent advances in biotechnology and the understanding of the genomics of natural product biosynthesis may enable the efficient production of the same bioactive molecules from plant-cell cultures and genetically engineered microorganisms [19], such as the large-scale microbial production of the artemisinin precursors amorpha-4,11-diene and artemisinic acid from genetically modified *Saccharomyces cerevisiae* and *Escherichia coli*[20]. Sources that are known to be rich in secondary molecules active against other diseases could also be targeted as potential sources of anti-malarial compounds. For example, the study of marine actinomycetes, long recognized as a rich source of secondary metabolites with anticancer activity, yielded the highly potent anti-malarial compound salinosporamide A (Figure 1), isolated from the marine actinomycete *Salinispora tropica*[21].

**Figure 2:**
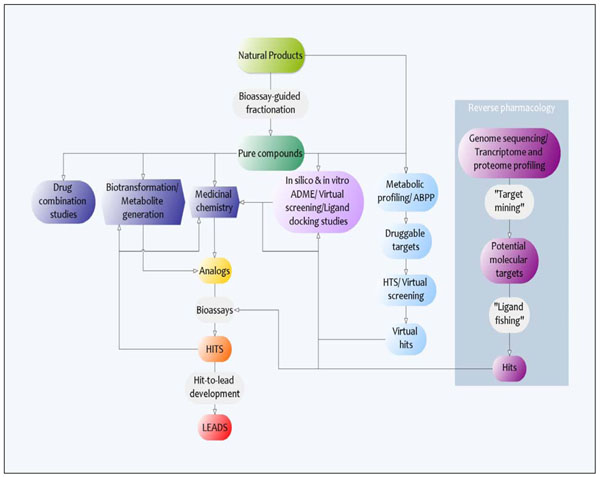
Development of leads from natural products.

However, it is noteworthy that most anti-malarial compounds isolated from natural sources are usually only moderately active, or possess challenging physicochemical and biological properties, and as such represent ‘hits’ rather than actual lead drug candidates. It, therefore, becomes apparent that considerable effort needs to be made to complement conventional natural product drug discovery and boost the chances of the successful identification of quality lead compounds from natural products, including their development from naturally derived hits. Such efforts must include the incorporation of what may be considered as more modern drug discovery strategies into anti-malarial natural product drug discovery endeavors (Figure 2). This would arguably increase the chances of successful identification and/or design of new potential leads, as well as considerably enhance the range and depth of knowledge and information that is derived from these discovery efforts.

With past and recent examples, the potential of some of these approaches is highlighted.

## Drug combinations

As mentioned earlier, one of the strategies for evading the development of resistance to anti-malarials is the use of combination therapies, which basically involves the co-administration of two or more anti-malarial agents. Drugs selected for combination in this way usually have a demonstrated synergistic effect when applied together, thereby allowing for the use of lower doses and achievement of more rapid therapeutic outcomes while taking advantage of the lower risk of the concurrent development of resistance by the microorganism to the co-administered agents. Although the emphasis is currently on artemisinin-based combination therapy (ACT) [5-7], the identification of new and efficacious combinations remains a priority, and the identification of new, active, naturally-derived compounds could provide additional possibilities. The potential of combinations of established anti-malarial drugs with other bioactive compounds derived from natural sources has already been demonstrated experimentally. For example, the combination of chloroquine and a mixture of febrifugine and isofebrifugine (isolated from the leaves of *Hydrangea macrophylla*) was found to be more active *in vivo* than the individual components of the combination [22].

## Dual drugs

Dual drugs, also known as hybrid compounds, refer to single chemical entities that consist of two drugs/active compounds/pharmacophoric units linked together covalently by a linker [23]. This aims to take advantage of the observed (or anticipated) synergistic or additive pharmacological activities of the hybrid components and enable the identification of highly active novel chemical entities. Highly active trioxaquines have been developed by covalently linking a 1,2,4-trioxane motif (a peroxide entity derived from the highly active natural sesquiterpene artemisinin) to a 4-aminoquinoline moiety (borrowed from chloroquine and other chloroquinoline-based anti-malarials) *via* an appropriate spacer. Several of these trioxaquine derivatives (Figure 1) were found to be extremely potent anti-plasmodial agents [24].

## Metabolism and metabolite identification studies

*In silico* (computational) and *in vitro* metabolism studies are primarily aimed at assessing the metabolic stability of promising hit and lead compounds, and are usually geared at providing useful information to guide and/or help in the interpretation of the results from subsequent pharmacokinetic studies and *in vivo* assays. In addition, such information also enables the drug discovery scientists to design and synthesize analogs of the primary compound(s) that are potentially more stable to metabolism and are likely to have improved oral bioavailability profiles [25].

However, and probably just as importantly, the *in vitro* generation and identification of primary metabolites of a compound can be an effective way of generating chemical diversity and augmenting the efforts aimed at identifying novel active compounds based on an identified hit. Pharmacologically active metabolites have been found to contribute significantly to the overall observed *in vivo* anti-malarial activity of many compounds. For example, N-desethylchloroquine, the primary metabolite of chloroquine, exhibits potent anti-malarial activity comparable to that of chloroquine [26,27]. This concept of pharmacologically active metabolites is just as applicable to anti-malarial hits derived from natural sources. Oshima and co-workers [28] reported the isolation and structural elucidation of two primary metabolites of febrifugine after its incubation for 1 hour at 37°C with mouse liver S9. Febrifugine is a highly potent anti-malarial alkaloid isolated from the roots, stem and leaves of the plant *Dichroa febrifuga*, a Chinese herb locally referred to as *Chang Shan*[29]. One of the major metabolites designated feb-A (Figure 1) showed extremely potent *in vitro* anti-plasmodial activity. Isofeb-A (Figure 1), a synthetic analog of feb-A, was even more potent than febrifugine *in vitro*, and had a significantly higher selectivity index [28].

In addition, metabolism studies may be useful in understanding the *in vivo* toxicity of a particular compound; this is because any toxic effects observed after the administration of a particular compound could be due to the generation of toxic metabolites. Such information derived from metabolism studies would then be crucial in the design of potent compounds devoid of the unwanted toxicity of the parent compound. Data from SAR, toxicity and metabolism studies of febrifugine was used to design a series of febrifugine analogs with the aim of identifying compounds with attenuated hepatotoxicity. This toxicity has precluded the development of febrifugine as a potential clinical drug, and is mediated by its arene oxide metabolite (Figure 1). Several analogs were identified that retained potent anti-plasmodial activity *in vitro* but were considerably less cytotoxic than febrifugine based on an *in vitro* cytotoxicity assay on rat hepatocytes. [30,31].

## Molecular modeling and docking tools

The use of modern molecular modeling and docking tools may be applied to enrich the process of derivatization of natural products by guiding or complementing the rational design and selection of more promising derivatives for subsequent synthesis and biological evaluation. An example of such a tool has been developed and validated for the analysis of artemisinin-based analogs as potential haem inhibitors [32].

## Natural product-derived pharmacophores and template-based virtual screening

Upon the elucidation of the structure of a novel anti-malarial compound from natural sources, and possibly informed by preliminary SAR data, it may be possible to identify its basic pharmacophoric unit(s). This not only facilitates more elaborate SAR studies, but also allows for the systematic searches of databases and libraries of known compounds (from both natural and synthetic sources) for structural analogs. Analogs identified in this way can then be acquired/ synthesized and assayed alongside the primary compound, and thereby provide additional SAR information and possibly even lead to the identification of additional active compounds that would have otherwise remained unidentified. The piperidinyl-(acetonyl)quinazoline moiety, a structural unit derived from febrifugine, was used as a template in the search for analogs in the Walter Reed Army Institute of Research (WRAIR) Chemical Information System (CIS) database. In this way, potent analogs of febrifugine were identified that also had significantly reduced *in vitro* cytotoxicity against a variety of mammalian cell lines [33].

## Natural product databases

An impressively large number of novel naturally-derived compounds have been isolated and characterized over the years. The creation of virtual and/or physical repositories of these compounds would help to keep track of these compounds and simultaneously create a rich resource that can be tapped for drug discovery efforts. Examples of such databases already exist [34,35], and these can be valuable for fragment- or ligand-based virtual screening, as well as for ligand docking studies on a variety of known protein targets. Physical repositories of crude and purified natural products can provide compound libraries for HTS, metabolic profiling, reverse pharmacology and related endeavors.

A recent example of the successful application of such a library of natural products and natural product-like molecules is in the discovery of highly active anti-malarial spiroindolone compounds. The identification of this class of compounds began with the screening of about 12,000 pure natural products and synthetic compounds with structural features found in natural products. Hits from this initial screen were then filtered based on *in vitro* anti-plasmodial activity, cytotoxicity against mammalian cells, physicochemical and pharmacokinetic properties. A compound related to the spiroazepineindole class was thereby identified as a starting point for medicinal chemistry efforts that eventually led to the identification of the highly active compound NITD609 (Figure 1). This compound was found to be as effective as artesunate, showed no evidence of diminished potency against drug-resistant strains, exhibited favorable *in vitro* solubility and permeability and did not show cytotoxicity across several human cell lines [36,37].

Physical repositories of natural products, such as the one set up by the Defense Science and Technology Organization in Australia, also offer a resource for anti-malarial screening. Screening of some of the extracts from this library revealed that some extracts, particularly those from the *Grevillea* species, possessed quite strong anti-plasmodial activity [38].

## Target-identification and reverse pharmacology

In addition to serving as a source of anti-malarial lead compounds, natural products can also add value to other techniques available for anti-malarial drug discovery. This is particularly with respect to the identification of new anti-malarial drug targets.

Metabolic profiling offers an exciting approach to the identification of (oftentimes new) molecular targets of biologically active molecules, and involves the use of tools such as NMR [39,40] and Mass spectroscopy [41,42] in the assessment of the metabolic response of an organism/cell/parasite following exposure to a bioactive molecule [43]. Bioactive natural products, by virtue of their chemical diversity, are particularly suited to act as probes for the identification of new molecular targets in this way [43,44]. The metabolic profiling of the malaria parasite has been reported [39,40,45], which would suggest the latent potential of this approach.

Another approach by which natural products may be useful in the identification of druggable targets is Activity-Based Protein Profiling (ABPP) [46]. Protein-reactive natural products (or privileged structures derived from them) can be useful in the development of ABPP probes [47]. For example, compound E-64, a cysteine protease inhibitor isolated from cultures of *Aspergillus japonicus*[48], is known to interact with plasmodial cysteine proteases [49] and has been successfully applied as a scaffold for the development of ABPP probes [50]. Proteins/enzymes identified in this way can then be investigated further as potential therapeutic targets by both classical mechanism of action studies or reverse pharmacology [51].

Reverse pharmacology offers an alternative means by which the molecular targets of biologically active compounds may be identified. Rather than apply a bioactive molecule in the identification of a molecular target, reverse pharmacology begins with the initial identification of potential protein targets (such as enzymes and receptors) by the application of bioinformatics tools that exploit the vast DNA-sequence databases provided by intensive genomic research. The potential targets identified in this way are then cloned and used to screen candidate ligands, which may include natural products [52]. As far as anti-malarial drug discovery is concerned, this approach has been rendered quite feasible by the successful sequencing of the genome of the human malaria parasite *P. falciparum*[53], now available as a database for vaccine development and drug discovery applications [54].

It has been argued that libraries of natural products and their derivatives are actually better suited for such screens simply because they span a much broader chemical space than most synthetic compound libraries currently available for this purpose [55]. It has even been proposed that screening libraries should be intentionally optimized by adding to them molecules with biogenic or natural-product-like scaffolds [56], further underlining the value of natural products to such strategies.

## Conclusion

Natural products may be lead compounds in themselves, or more likely may serve as hits that may be useful in providing pharmacophores/templates that can guide the design of potentially superior analogs and/or the mining of existing databases of synthetic and semi-synthetic compounds for previously untested and potentially active analogs. Promising analogs designed or identified in this way may be expanded and developed further by introduction of (bio)isosteric substitutions suggested by scaffold hopping techniques, or by the application of *in silico* (computational) and *in vitro* profiling of physicochemical and ADME(T) parameters to enable the rational design of potentially improved/superior analogs. The *in vitro* generation of metabolites can ably complement synthetic and semi-synthetic efforts at derivatization of primary, naturally derived, bioactive molecules, thereby broadening the chemical diversity around these molecules and increasing the likelihood of identifying promising compounds. Natural products can also find wide application in target-identification studies - metabolic profiling of the malaria parasite has been reported and the possible role of natural products acknowledged; ABPP probes based on scaffolds known to interact with malarial proteins have been developed; the elucidation and reporting of the malaria genome affords the opportunity for reverse pharmacology.

The potential of natural products to provide or inspire the development of anti-malarial lead compounds is, therefore, really quite evident. However, to raise the chances of the actual realization of this potential, it has become necessary to think beyond the confines of conventional natural-product drug discovery. The application of a wide variety of scientific tools and the close and interactive collaboration of experts in diverse scientific disciplines (such as chemistry, pharmacology, molecular biology and genetics) has become practically obligatory if these truly multi-disciplinary efforts are to indeed be successful. The fact that literature on the application of some of these approaches towards anti-malarial drug discovery based on natural products is sparse is indicative of their underutilization in this regard, a situation that should arguably be addressed.

## Competing interests

The authors declare that they have no competing interests.
